# The Microbiota/Microbiome and the Gut–Brain Axis: How Much Do They Matter in Psychiatry?

**DOI:** 10.3390/life11080760

**Published:** 2021-07-28

**Authors:** Donatella Marazziti, Beatrice Buccianelli, Stefania Palermo, Elisabetta Parra, Alessandro Arone, Maria Francesca Beatino, Lucia Massa, Barbara Carpita, Filippo M. Barberi, Federico Mucci, Liliana Dell’Osso

**Affiliations:** 1 Department of Clinical and Experimental Medicine Section of Psychiatry, University of Pisa, 56100 Pisa, Italy; beatriceb.90@live.com (B.B.); stefania.palermo@hotmail.com (S.P.); elisabettaparra@virgilio.it (E.P.); alessandroarone@libero.it (A.A.); mariaf.beatino@gmail.com (M.F.B.); massa.lucia116@gmail.com (L.M.); barbara.carpita1986@gmail.com (B.C.); fil.barberi@gmail.com (F.M.B.); liliana.dellosso@med.unipi.it (L.D.); 2 Unicamillus—Saint Camillus International University of Medical and Health Sciences, 00131 Rome, Italy; 3 Dipartimento di Biochimica e Biologia Molecolare, University of Siena, 53100 Siena, Italy; federico.mucci@med.unipi.it

**Keywords:** microbiota, gut–brain axis, central nervous system, immune system, autism spectrum disorders, mood disorders, obsessive-compulsive disorder, schizophrenia, novel psychotropic drugs, neuropsychiatric disorders

## Abstract

The functioning of the central nervous system (CNS) is the result of the constant integration of bidirectional messages between the brain and peripheral organs, together with their connections with the environment. Despite the anatomical separation, gut microbiota, i.e., the microorganisms colonising the gastrointestinal tract, is highly related to the CNS through the so-called “gut–brain axis”. The aim of this paper was to review and comment on the current literature on the role of the intestinal microbiota and the gut–brain axis in some common neuropsychiatric conditions. The recent literature indicates that the gut microbiota may affect brain functions through endocrine and metabolic pathways, antibody production and the enteric network while supporting its possible role in the onset and maintenance of several neuropsychiatric disorders, neurodevelopment and neurodegenerative disorders. Alterations in the gut microbiota composition were observed in mood disorders and autism spectrum disorders and, apparently to a lesser extent, even in obsessive-compulsive disorder (OCD) and related conditions, as well as in schizophrenia. Therefore, gut microbiota might represent an interesting field of research for a better understanding of the pathophysiology of common neuropsychiatric disorders and possibly as a target for the development of innovative treatments that some authors have already labelled “psychobiotics”.

## 1. Introduction

The terms “microbiota” and microbiome refer, respectively, to the collection of bacteria, viruses and fungi colonising different parts of the body, and to the complete genetic material encoded by the microbiota [1,2,3]. The gut microbiota, i.e., the commensal microorganisms within the gut, performs essential tasks for the normal functioning of the organism, such as the fermentation and digestion of carbohydrates, development of lymphoid tissues associated with the mucous membranes, production of vitamins, prevention of colonisation by pathogenic microorganisms and stimulation of the immune system [2,4,5,6]. The bacterial cells forming intestinal microbiota outnumber human cells by 10 times and encode for a gene set that is 150 times larger than the human one [1]. The human gut microbiota, mainly consisting of Proteobacterias, Firmicutes, Actinobacteria and Bacteroidetes, changes during the course of life, as it is constantly influenced by several individual factors, such as the type of birth, infections, therapies, diet, smoking, physical activity, stressful events, environmental factors and medical diseases [7,8,9]. It is also worth highlighting that the brain’s development, depending on pre- and post-natal genetic and environmental factors, occurs in parallel with the constitution of the microbiota. A newborn’s microbiota has a low density but, as the individual grows, it is enriched with certain microorganisms, becoming increasingly capable of activating signals and metabolic pathways that modulate neuronal function [10,11,12,13].

The development and functioning of the central nervous system (CNS) depend on the integration of central factors, peripheral signals and environmental influences. The gut microbiota represents an excellent example of this integrated system, as it is an important link between our body and the environment. Again, given that it engages in two-way communication with the CNS, it outlines a fascinating model of the connection between central and peripheral systems [3].

The gastrointestinal (GI) system is regulated by the so-called enteric nervous system (ENS), which is formed by neurons located in the GI tract itself and enteric glial cells [6,14]. This results in bidirectional communication between the brain and gut. The gut and brain influence each other through different mechanisms that are mediated by neurotransmitters and immune modulators, such as cytokines and metabolic products and hormones, with a pivotal involvement of the hypothalamic–pituitary–adrenal (HPA) axis [15]. The gut microbiota seems to play a major role in this mutual connection that is called the “gut–brain axis” [16]. Gut microbiota is capable of producing and modulating the bioavailability of the main neurotransmitters, influencing GI motility and fermenting dietary polysaccharides, that is to say, the gut microbiota seems to influence important processes that are often altered in different neuropsychiatric conditions, such as epilepsy, stroke, Parkinson’s disease, schizophrenia (SZ), obsessive-compulsive disorder (OCD), depression, anorexia nervosa (AN) and behavioural and neurodevelopmental disorders [17,18,19,20,21,22,23,24,25]. Therefore, dysbiosis, i.e., the imbalance in the composition of microbiota, might be one of the factors involved in the onset and maintenance of both some psychiatric and functional GI disorders. It is not uncommon to observe inflammatory bowel disease (IBD) in subjects suffering from mood disorders (MDs), anxiety and OCD, or abdominal pain in patients with SZ or panic disorder [26,27,28,29].

The immune system appears to be at the heart of the gut–microbiota–brain relationship. Indeed, an altered composition of the gut microbiota might compromise the epithelial intestinal integrity and lead to a defective defence against pathogenic microorganisms, with consequent inflammatory reactions and, ultimately, neuro-inflammation [30]. Moreover, dysbiosis causes an increase in the amount of short-chain fatty acids (SCFAs), such as acetate, propionate and butyrate, that might activate microglia cells, i.e., the immune cells of the CNS, leading to an increase in cytokines that may eventually alter brain connections and the blood–brain barrier (BBB) [31,32]. Interestingly, microbial-induced BBB dysfunction is hypothesised to play a causative role in mood and anxiety disorders, SZ, autism spectrum disorders (ASDs) and neurodegenerative diseases [33]. A role of the gut–brain axis in the development of CNS tumours was also proposed [34]. Finally, a growing number of findings suggest that the microbiota might modulate neuronal maturation and myelination processes in brain areas that are responsible for the control of emotions, executive functions and working memory, which are impaired in SZ, MDs and ASDs [35,36,37].

Given the available evidence, it is plausible that a better understanding of the influence exerted by intestinal flora on the CNS and the role of the gut microbiota in the onset and maintenance of psychiatric disorders might lead to producing novel treatments, including probiotics, personalised lifestyles, faecal microbiota transplantation (FMT) and specific diets [38].

Therefore, the aim of this paper was to review and comment on the current literature on the role of the intestinal microbiota and gut–brain axis in some common neuropsychiatric conditions.

## 2. Methods

According to the PRISMA guidelines [39], the databases of PubMed, Scopus, Embase, PsycINFO and Google Scholar were accessed in order to research and collect English language papers published between 1 January 1969 and 15 May 2021. Free text terms and MeSH headings were combined as follows: “(Microbiota OR gut microbiome OR gut-brain axis) AND (CNS OR psychiatric disorders OR neuro-inflammation OR immune system OR depressive disorder OR depression OR mood disorders OR bipolar disorder OR obsessive-compulsive disorder OR OCD OR schizophrenia OR neurodevelopmental disorders OR eating disorders OR autism)”. All the authors agreed to include conference abstracts, posters and case reports in the review if they were published in indexed journals. The following inclusion criteria were adopted: studies carried out in clinical samples of adults and children/adolescents, reliable diagnosis of psychiatric disorders according to structured interviews and standardised criteria and reliable assessment of outcome measures. All the authors equally contributed to identifying potential information specific to this topic amongst the titles and abstracts of the publications.

## 3. Results

**Figure 1:**
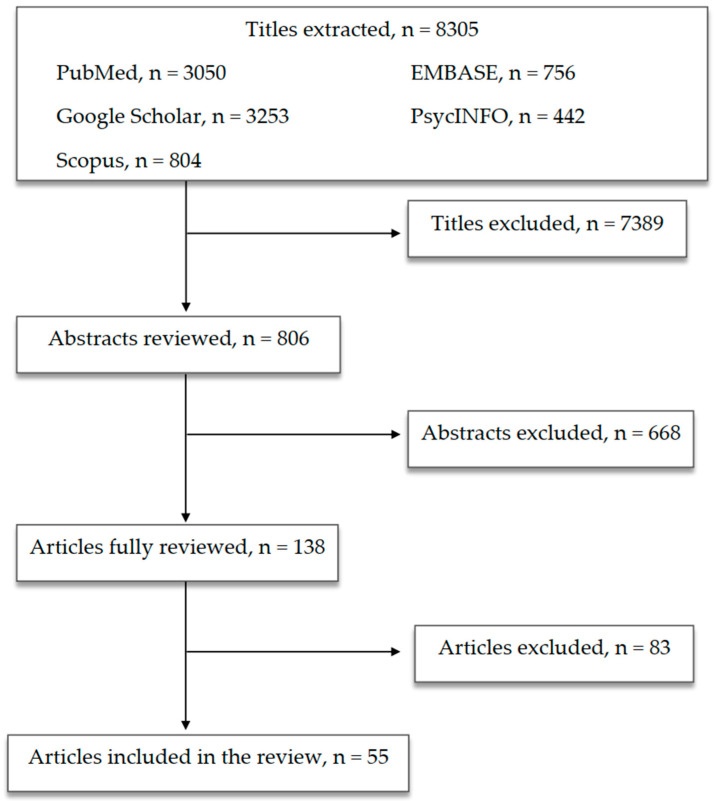
Article selection flowchart.

The first selection excluded 7389 titles because they were: duplicates or duplicated results, not related to the scope of the paper or not informative enough. The second selection excluded 668 abstracts after being read and reviewed, as the information reported did not fulfill the scope of our paper and/or the presented information did not seem relevant to the discussed topic. Subsequently, 83 publications were excluded after being completely read and evaluated, as they did not provide enough information and/or were not sufficiently in line with our review. Finally, 55 papers were included in the present review (Figure 1).

## 4. CNS and Microbiota: The Gut–Brain Axis

The large number of novel studies on the relationships between microbiota and the CNS has led to the recognition of the gut–brain axis, that is to say, the bidirectional connection occurring between the gut microbiota and the brain through hormonal, metabolic, immunological and neural signalling, with the latter involving central, autonomic and enteric nervous systems [6,40,41,42]. This mutual connection seems to reflect a reciprocal influence: the diversity in microbiota composition affects brain development and behaviours, and vice versa [42].

To date, the information on bottom-up regulation (i.e., the influence of gut microbiota on the brain) mainly derives from translational and animal model studies, with a particular focus on anxiety and depression, while studies in humans are still limited [43]. Germ-free (GF) mice, i.e., mice without commensal intestinal bacteria, showed a reduction in anxiety-like behaviours [44,45,46], while according to another study, GF mice showed deficits in social cognition, anxiety-like behaviours and altered stress response, which was maybe related to a bigger volume of amygdala and hippocampus and a different morphology of dendrites in these brain regions in comparison to conventionally colonised (CC) mice [47]. Puppies born from GF mice colonised with fast-growing human neonatal microbiota showed accelerated neuronal differentiation and fewer signs of inflammation than those colonised with slow-growing human microbiota [48].

Interestingly, brain changes that are promoted by microbiota might occur through the regulation of gene expression and neuronal transcription [48,49]. A murine model study demonstrated the upregulation of myelin-related genes in GF mice, specifically in the prefrontal cortex (PFC), leading to hypermyelinated axons. Furthermore, the subsequent colonisation of these animals (the so-called exGF) resulted in a reverted modulation [49]. Gene expression regulation that was driven by intestinal microbiota also led to the modulation of neuro-inflammation, production of insulin-like growth factor-1 (IGF-1) and changes in multiple neurotransmitter (serotonin (5-hydroxytryptamine, 5-HT), dopamine, glutamate and gamma-aminobutyric acid (GABA)) pathways, transporters and ion channels [48]. Focusing on neurotransmitters, male GF mice show increased 5-HT and 5-hydroxyindoleacetic acid (5-HIAA, the main 5-HT metabolite) in the hippocampus [46], while Bifidobacterium infantis administration in rats increased tryptophan, the 5-HT precursor [50]. As already mentioned, the effects of gut microbiota on neurotransmission extend beyond 5-HT. Non-pathogenic bacteria, such as Lactobacillus rhamnosus, modulate GABAergic transmission in mice, with beneficial effects on anxiety and depression [51], and GABA production by cultured intestinal strains of Lactobacillus and Bifidobacterium was observed [52]. Nonetheless, regarding the relationship between brain and GI tract, it is worth noting that about 90% of 5-HT is synthesised in the gut, where it modulates GI motility, and then is sequestered by platelets and transported to various body sites, acting as a pleiotropic hormone [53,54]. Indeed, the intestinal synthesis of 5-HT seems to be positively influenced by microbiota, consequently increasing 5-HT in the GI mucosa and lumen, platelets, blood and brain. As such, microbiota influence peripheral and central 5-HT concentrations [54].

Stress is another factor involved in this complex system. The bidirectionality of the gut–brain axis includes a top-down modulation, that is to say, the modulation of GI functions and permeability itself is influenced by psychological stress, which often serves as a trigger for the onset, relapses and recurrences and worsening of psychiatric disorders [42,55]. Indeed, some studies in animal models showed that stressed pups had higher plasmatic corticosterone levels, enhanced systemic immune responses and altered microbiotas [56,57,58]. The HPA axis is activated by inflammatory cytokines and other products, including bacterial ones, as shown in infections sustained by Escherichia coli, a member of the Enterobacteriaceae family, i.e., bacteria colonising the enteric system [59,60,61,62].

Furthermore, the links between the brain and gut also play a role in the immune response. An example of this link is provided by microglia. As the resident macrophages of CNS, microglia are involved in the immune surveillance of the CNS itself [63], and as such, possibly in different brain disorders [64,65]. Microglia maturation, activation and function are affected by microbiota composition. According to some authors, microglia changes are driven by gut eradication, re-colonisation and variations in microbiota complexity. Interestingly, GF mice share defective microglia and impaired innate immunity [64].

It should be noted that the GI tract represents the largest immune organ, as well as the largest surface of contact with external agents [55]; therefore, it was hypothesised that alterations of intestinal flora, through regulatory T cells (Treg) abnormalities, might be involved in the epidemic of allergic, inflammatory and autoimmune diseases and also in psychiatric disorders [66,67,68,69,70,71,72,73].

The gut microbiota also contributes to maintaining the integrity of the intestinal barrier. Dysbiosis increases the permeability of this barrier (the so-called “leaky gut” syndrome), allowing for bacterial translocation and the passage of microbial products and inflammation mediators into the bloodstream, and eventually in the CNS, triggering an inflammatory reaction [74,75,76,77]. Furthermore, according to other studies, the microbiota also influences the permeability of the BBB. Indeed, GF mice display increased BBB permeability compared with pathogen-free mice due to a diminished expression of tight junction (TJ) proteins (occludin and claudin-5). The exposure of GF mice to pathogen-free microbiota leads to a higher expression of TJ proteins and a decreased BBB permeability [78]. The model of the antibiotic-induced gut dysbiosis was also explored, as it would cause changes in the expression of TJs, cytokines, brain-derived neurotrophic factor (BDNF) and 5-HT transporter, eventually resulting in cognitive impairment [79]. Therefore, gut flora has been hypothesised to be involved in both “leaky gut” and “leaky brain” syndromes [80].

## 5. Microbiota and Psychiatric Disorders

Recently, an increasing amount of studies have been focusing on how the interactions between microbiota and CNS might play a role in the pathophysiology of neuropsychiatric disorders, mostly MDs, OCD, neurodevelopmental disorders (especially ASDs) and neurodegenerative diseases. Therefore, the therapeutic potential of microbiota-targeted treatments was proposed to the extent that some authors proposed to call them “psychobiotics” [43,81,82,83,84,85,86,87,88].

Indeed, the interactions between the host and its microbiota seem to be able to produce significant changes in brain networks, thus influencing behaviours and neuropsychiatric disorders [89].

Taking into account the immunological model for psychiatric disorders, the gut microbiota’s composition might influence psychic functions to the extent that the inflammatory cascade and the immune stimulation vary depending on the bacterial species involved [62]. Nonetheless, according to this model, gut microbiota might also be one of the mediators responsible for the well-known relationship between psychiatric disorders and GI symptoms and disturbances [6].

### 5.1. Mood Disorders

Pervasive dysregulation of mood and psychomotricity, alterations of biorhythms, changes in appetite and sleep pattern, cognitive disturbances and impaired global functioning characterise MDs. Currently, this nosological category includes major depression (MDD), bipolar disorder (BD) and dysthymia [90]. While patients with MDD only suffer from depressive episodes, mood fluctuations of both polarities are typical of BD, that can be distinguished in BD of type I (BDI) when there is at least one lifetime manic episode or BD of type II (BDII) when depressive episodes alternate with hypomanic ones [90].

The aetiology of MDs is largely unclear and is still the subject of deep investigation. According to the most comprehensive hypotheses, MDD results from the interaction between an individual vulnerability and a variety of stressors/triggers entailing anatomic, physiologic and neurochemical modifications [91,92,93,94,95,96,97]. Besides the classical biomarkers that have been widely described in the past few decades, it is now evident that they are part of a more complex picture involving inflammatory/immune systems dysfunctions [98,99,100,101,102], up to the point that MDD is considered a systemic disease [103,104]. Basically, different intestinal bacteria influence the metabolism of neurotransmitters, by modifying the availability of tryptophan and tyrosine and, consequently, 5-HT and dopamine, respectively [105]. Not surprisingly, the pathophysiological role of dysbiosis and the subsequent mild inflammatory state in the onset and evolution of MDD was widely described, together with changes in gut microbiota composition [106,107,108,109,110,111,112,113]. It was hypothesised that an altered intestinal permeability might facilitate the presence of circulating cytokines. Moreover, high serum levels of IgM and IgA against Gram-negative lipopolysaccharide (LPS) were found in depressed patients, suggesting that an increased intestinal permeability allows enterobacteria to trigger infections [114,115]. The relationship between microbiota and mood alterations has long been investigated in an attempt to assess differences between microbiota composition in patients suffering from MDs and healthy controls [116]. A shotgun metagenomic method was used to investigate 156 faecal samples from depressed patients and 155 faecal samples from controls [117]. The results showed some differences in viruses, bacteria and metabolites, but not in protozoa and fungi. Depressed patients showed a greater amount of bacteria belonging to the genus Bacteroides, which were capable of inducing the production of cytokines and mediating inflammatory responses [118,119], as well as a reduction in bacteria of the genera Eubacterium and Blautia, with the latter showing anti-inflammatory properties [120]. It was hypothesised that bacterial production of GABA can reduce depressive symptoms, with intestinal levels of GABA influencing brain functions. Indeed, low levels of GABA and its metabolites were found in the faeces of depressed subjects, as well as a reduction in microbes that are capable of degrading phenylalanine. Interestingly, patients suffering from depression seem to also show downregulation of the BetB gene, that is involved in the metabolism of arginine into GABA [121,122,123].

Depressed subjects, as well as those suffering from IBD and chronic fatigue syndrome, show higher levels of Alistipes, a bacterium belonging to the phylum Bacteroidetes. Increased permeability of the intestinal epithelium allows the passage of inflammation factors that are induced by this bacterium to pass into the bloodstream [58,124,125,126]. Interestingly, GF mice, after undergoing FMT from MDD patients, exhibit depression-like behaviours [108,116].

As compared with data in MDD, the literature on BD is more limited. Patients with BD show lower amounts of faecal Bifidobacterium, Lactobacillus and Faecalibacterium than healthy subjects [107,109]. As regards the fungal component of gut microbiota, Candida albicans IgG levels were significantly higher in male patients suffering from BD (and also from with SZ) than in control subjects [127].

According to some authors, the severity of manic symptoms seems to be related to the prescription of antibiotics [128]. This finding might be due to the fact that bacterial infections that require an antibiotics prescription might lead to an inflammatory response and immune activation that, in turn, would induce acute mania. Another possible explanation is that antibiotics might modify the microbiota’s composition, hence increasing the risk of altered mood states. Nonetheless, the high rate of bacterial infections (and, therefore, of antibiotics assumption) in manic individuals could reflect a decreased performance of their immune system [128] (Table 1).

### 5.2. Obsessive-Compulsive Disorder and Related Conditions

Obsessive-compulsive disorder (OCD) is a common psychiatric condition that is characterised by obsessions, compulsions or both. Obsessions are recurrent, persistent, intrusive and unwanted thoughts, urges or images that cause marked anxiety or distress. The individual tries to ignore, suppress or neutralise obsessions by performing a compulsion that is a repetitive behaviour or mental act [90].

Obsessive-compulsive disorder was included in the “anxiety disorders” group [130] until the publication of DSM-5, where it gained categorical autonomy within the “obsessive-compulsive and related disorders” (OCDRs) [90]. However, according to some authors, many of the findings on the relationship between microbiota and anxiety-like behaviours may also be related to OCD, given that anxiety remains a pivotal dimension in OCD [42]. Nonetheless, some attempts were made to clarify how gut microbiota alterations are specifically related to obsessive-compulsive symptoms.

Recent literature has mostly highlighted the role of the immune system, the intestinal microbiota and their interactions in the onset and maintenance of OCD. Taken together, the findings collected so far suggest that immunological dysfunctions and altered gut microbiota composition might be involved in the aetiology of OCD. The marble-burying test, a murine model for anxiety and OCD-like behaviours, was affected by gut microbiota manipulation [131,132,133]. RU 24969, a 5-HT1A-1B receptor agonist, was used in mice to induce OCD-like behaviours that were attenuated by pre-treatment with probiotics (Lactobacillus rhamnosus) and fluoxetine, a selective 5-HT reuptake inhibitor (SSRI) that is considered a first-line treatment of this condition [134], in comparison to pre-treatment with saline. Moreover, the protection against OC symptoms observed with probiotics and with fluoxetine pre-treatments was similar [132]. Similarly, quinpirole hydrochloride was injected in rats to induce OC symptoms that improved after treatment with Lactobacillus casei shirota, with fluoxetine and with the combination of both. These treatments also caused an increase in BDNF and a decrease in 5-HT2A receptor expression in the orbito-frontal cortex (OFC), one of the brain areas that is possibly altered in OCD [135].

A recent case report of a boy with ASD, OCD, tics, self-injurious behaviour (SIB), a history of GI disturbances and a global immune dysregulation documented that Saccharomyces boulardii administration, aimed at reducing GI symptoms, resulted in an improvement of OCD and SIB [136]. The authors also underlined how ASD, OCD and GI manifestations are often in comorbidities while suggesting a possible common pathophysiological role of altered gut microbiota [136]. Since converging reports highlight the role of the HPA axis and stress in OCD onset and worsening [137,138,139], alteration of the gut microbiota might represent the link between the stress response and the development of OCD [140]. As already mentioned, stressors may induce modifications in the gut microbiota populations [141], such as a decrease in Bacteroides and an increase in Clostridium species, and lead to bacterial translocation [57]. On the other hand, a randomised double-blind controlled trial reported that oral administration of Lactobacillus reduced salivary cortisol levels in young adults under examination stress [142].

It was suggested that even antibiotics might alter the composition of intestinal flora up to the extent that they and not group A beta-haemolytic streptococcus would be the causative factor of the paediatric autoimmune neuropsychiatric disorders associated with streptococcal infection, the so-called PANDAS [140,143], or, more recently, “paediatric acute-onset neuropsychiatric syndrome” (PANDAS) and “childhood Acute Neuropsychiatric Syndrome” (CANS) [140].

Further evidence of the relationships between microorganism colonisation and the immune system that might be useful regarding OCD (and other psychiatric disorders) derives from the observation of antimicrobial activity exerted in vitro by SSRIs alone and in combination with antibiotics, resulting in a decreased minimal inhibitory concentration (MIC) and the conversion of multiply resistant bacterial strains to sensitive ones [144] (Table 2).

### 5.3. Schizophrenia

Schizophrenia is a psychiatric disorder, usually with an early onset during adolescence, and is characterised by delusions, hallucinations, disorganised thinking (speech), grossly disorganised or abnormal motor behaviour (including catatonia) and negative symptoms, resulting in a severe impairment of global functioning and cognitive abilities [90,145]. The aetiology of SZ is multifactorial, as it includes the interaction between genetic and environmental factors [146]. Within the framework of this multifactorial model, the involvement of the immune system was also hypothesised based on some evidence showing that maternal infections during pregnancy increase the risk of psychosis [147] and that schizophrenic patients often suffer from comorbid autoimmune diseases or atopic disorders [148,149], as well as alterations of different inflammatory parameters [150,151]. Indeed, subjects with acute psychosis show high serum levels of IL-6, TNF-α and soluble IL-2 receptor (sIL-2R); chronic SZ patients have increased Il-6, IL-1β and IL-2R concentrations [152]; and those with a psychotic onset display high prostaglandin E2 (PGE2) levels and high COX activity [153,154]. According to genome-wide association studies [155], many of the 108 loci associated with susceptibility to developing SZ are expressed in tissues with immune activity and some human leukocyte antigen (HLA) loci are related to an increased likelihood of developing SZ [156]. Studies in animal models suggested that infections during pregnancy might affect brain development in the offspring through changes in microglia, leading to behavioural and cognitive alterations in adolescence [157].

Regarding the relationship between microbiota and SZ, research is still in its infancy. As mentioned above, animal studies underlined the role of microbiota in the postnatal development and maturation of neuronal, immune and endocrine systems, which influence processes, such as cognition and social behaviour, that are altered in SZ patients [158]. Studies conducted on schizophrenic patients led to intriguing results. Indeed, both treated and untreated patients with SZ showed altered gut microbiota and decreased microbiome heterogeneity compared with healthy controls. Moreover, some unique bacterial taxa and high Lactobacillus gut levels were related to the severity of the clinical picture in patients with SZ [159,160]. A cross-sectional study that analysed the composition of faecal microbiota in both schizophrenic and healthy subjects through 16S rRNA sequencing showed that the first showed abundances of the Proteobacteria Phylum, Succinivibrio, Megasphaera, Collinsella, Clostridium, Klebsiella and Methanobrevibacter. Therefore, the authors proposed a microbiota-based diagnosis and prognosis of SZ [161]. A study conducted on first-episode schizophrenic patients reported altered microbiota composition that was significantly modulated by risperidone, a first-generation antipsychotic (FGA), an effect possibly related to drug-induced metabolic changes [162]. Further evidence suggests that antipsychotics may indeed affect microbiota levels in patients with SZ, specifically in regard to the taxonomic distribution in the case of chronic treatments [163]. The effects of antipsychotic may also be boosted by some antibiotics, such as minocycline, which are able to modify the gut microbiota [164]. However, evidence on this matter is still controversial, as different studies did not detect similar effects of APs in the modulation of gut microbiota [165,166]. Again, the gut microbiota has been proposed as a factor that is responsible for the lack of response observed in some schizophrenic patients [167]. On the other hand, it was pointed out how probiotics showed no clinical utility in both negative or positive symptoms, albeit only three studies were fully reviewed [168]. Interestingly, in a murine model, inulin, which is a dietary fibre mainly produced by plants [169], was also proposed as a potential treatment in SZ patients due to its anti-inflammatory action and the effects exerted on the gut microbiota [170].

Recently, the relationship between the gut microbiome and brain morphological and functional correlates was investigated in patients with SZ. At the genus level, compared to healthy control subjects, SZ patients displayed a higher abundance of Veillonella, whilst the abundance of Roseburia and Ruminococcus was lower. Moreover, a comparison of MRI images highlighted significant differences in both the volume of gray matter and the regional homogeneity amongst the two groups and higher amplitudes of low-frequency fluctuation in SZ patients. Finally, both changes in gray matter volume and regional homogeneity correlated with the diversity of the gut microbiota [171]. In a similar fashion, significant changes in the volume of the right middle frontal gyrus seem to be related to the specific composition of gut microbiota in SZ [163]. Besides the hypothesis stating that altered gut microbiota might cause the abnormal activation of the immune system, making the gut barrier more susceptible to micro-environmental changes and leading to neuro-inflammation processes involving microglia-mediated neuronal damage, apoptosis, abnormal brain development and altered connectivity between brain regions, even epigenetic modulation might be a mechanism underlying the link between microbiota and SZ [172]. Indeed, gut microbiota might affect gene expression through acetylation and methylation processes in response to environmental cues, possibly constituting a link between environmental risk factors and epigenetic changes [173,174].

Taken together, these findings, albeit limited, appear intriguing. However, more studies are needed to clarify the role of gut microbiota in SZ in order to increase the pathophysiological mechanisms of this disorder and, eventually, to promote and improve therapeutic strategies (Table 3).

### 5.4. Autism Spectrum Disorders

Autism spectrum disorders (ASDs) include different psychopathological conditions that are characterised by persistent deficits in social communication and social interaction, as well as limited and repetitive behaviours, interests or activities. According to DSM-5 [90], ASDs include autistic disorder, Asperger’s syndrome, childhood disintegrative and pervasive developmental disorders not otherwise specified [90].

Subjects with autism are often reported to suffer from GI symptoms [175,176,177,178,179,180]. A systematic meta-analysis found a significantly higher prevalence of GI symptoms amongst ASD children compared to control subjects [181]. According to some authors, these GI symptoms even correlate with autism severity [182,183]. Along with GI symptoms, ASD subjects were found to also show an altered gut flora [184,185,186].

In the last two decades, an impressive number of cross-sectional studies reported significant differences in microbiota composition between children with an ASD and controls [187,188,189,190,191,192,193,194,195], thus strengthening the hypothesis of a possible link between GI dysbiosis and ASD. On the other hand, a cross-sectional study comparing intestinal microbiota of autistic children (with and without GI symptoms) and their siblings detected no significant intergroup differences [196]. The authors then suggested that GI symptoms in ASD might depend on anxiety and diet patterns, rather than on microbiota alterations. Indeed, it is well known that ASD is frequently associated with peculiar eating patterns, usually characterised by food selectivity [197,198] and avoidant/restrictive food intake disorder, which sometimes may lead to nutritional deficiency diseases [199]. Due to this evidence, when it comes to investigating microbiota alterations in ASDs, it was recommended that more studies considering the eating habits of participants be undertaken [87].

Other authors also wonder whether altered microbiota in ASD represents a comorbid condition, a causative factor or a consequence of the neuropsychiatric disorder [187,200]. In any case, the large number of studies documenting the possible involvement of microbiota in ASD pathogenesis led to considering whether treatments acting on gut flora could ameliorate ASD symptoms. An open-label trial consisting of a 12-week administration of vancomycin (a minimally absorbed oral antibiotic) in 11 children with regressive-onset autism reported behavioural improvement; however, this was not sustained at follow-ups that occurred between 2 and 8 months later [201]. In another open-label trial, Kang et al. tested the effects of microbiota transfer therapy (MTT) in 18 children with ASD [183]. MTT consisted of a 2-week antibiotic treatment, a bowel cleanse and then faecal microbiota transplant (FMT). At the end of the treatment, there were changes in microbiota composition (in particular, an increase in Bifidobacterium, Prevotella and Desulfovibrio), an 80% reduction of GI symptoms and improvement of ASD symptoms. All the results were confirmed after 8 weeks [183] (Table 4).

### 5.5. Miscellanea

The gut–brain axis appears to be involved in several other different neuropsychiatric syndromes in children and adults that will be briefly reviewed herein for completeness, although the available data are still limited. 

Attention-deficit/hyperactivity disorder (ADHD) is a neurodevelopmental disorder that is characterised by inappropriate levels of hyperactivity, difficulty in controlling behaviour and/or attention problems [90]. A link between microbiota and ADHD development or manifestations was suggested. Preliminary evidence indicates that specific diets or dietary components modulating gut microbiota might influence brain activity in regions involved in cognitive and behavioural processes that are relevant for ADHD symptoms [89,202] (Table 5).

Eating disorders (EDs) represent a major health concern, especially in Western countries and amongst the young population [203], and are characterised by a persistent disturbance of eating or eating-related behaviours, resulting in the altered consumption or absorption of food and leading to significant impairment in physical health or psychosocial functioning [90]. Anorexia nervosa (AN), bulimia nervosa (BN) and binge eating disorder (BED) are the three most relevant categories of EDs [203]. Subjects suffering from BN engage in recurrent episodes of binge eating and inappropriate compensatory behaviours aimed at preventing weight gain, while AN is characterised by a restriction of nutritional intake, with or without binge-eating/purging episodes, resulting in significantly low body weight. Both AN and BN share a misinterpreted experience of the individual’s body weight or shape, excessively influencing self-evaluation. Binge eating disorder is otherwise characterised by recurrent binge-eating episodes that are not associated with compensatory behaviours [90]. Some studies reported significantly altered microbiota, such as reduced diversity and taxa abundance, possibly due to starvation, in patients with AN. For this reason, nutritional strategies and psychobiotics administration can become potentially relevant in AN treatment [204,205]. Even patients with BN and BED may show several GI symptoms. A few recent studies highlighted the role of the intestinal microbiota in the pathophysiology of these disorders, suggesting a possible adjuvant therapy to the psychopharmacological one [203,206].

Since more specific data on these disorders are lacking, more in-depth studies are warranted to better understand the possible links between gut microbiota and EDs (Table 6).

## 6. Conclusions

The mounting evidence of connections between the brain and peripheral organs allowed for highlight the possible existence of fine-tuned reciprocal influences between the CNS and the gut microbiota. Given that the gut microbiota may affect brain functions through hormonal messengers and impact neurotransmitter metabolism and immune systems, it is not surprising that the gut microbiota was supposed to be involved in the pathophysiology of several neuropsychiatric disorders. 

The most consistent, albeit scattered findings are those gathered for MDs, specifically MDD and ASDs, while the information for BD, OCD, ADHD and EDs is still limited, and is mainly obtained through murine and translational models. 

In any case, the findings of altered gut composition in some conditions, although controversial, would suggest possible novel therapeutic targets. It is noteworthy that a recent review underlined how some antimicrobials show AD properties (incidentally the first drug proposed for depression treatment was isoniazid, a drug used for the treatment of tuberculosis), and how some SSRIs, such as sertraline and fluoxetine, show antimicrobial effects [129]. Therefore, it was proposed that these effects would represent another positive outcome when treating MDD [129]. However, it is conceivable that the same benefits might be obtained in all psychiatric disorders or symptoms targeted by ADs and characterised by gut microbiota dysbiosis, augmented gut permeability, bacterial translocation and neuro-inflammation.

Further controlled studies, possibly conducted in large clinical samples, are needed to deepen the role of microbiota in neuropsychiatry, as well as to explore the possible therapeutic role of anti-, pre- and pro-biotics, as well as FMT, at least in that non-negligible part of those patients who still do not respond to the available approved treatments. However, the association between dysbiosis and several other neuropsychiatric disorders seems to be highly probable, possibly allowing for the enrichment of psychopharmacological treatments with psychobiotics for an ever-increasing range of pathological conditions.

## Figures and Tables

**Table 1:** Studies on microbiota and mood disorders (MDs).

Authors and Year	Type of Study	Population	Methods	Findings
Mangiola et al., 2016 [84]	Review	-	Selected studies on the role of gut microbiota and the use of microbiota-modulating strategies in MDs/ASD	- Reduced (anxiety-like behaviour in GF mice after the restoration of the intestinal microbiota; Improved depression and anxiety symptoms in mice after the administration of probiotics; - Increased Alistipes in depressed patients; negative correlation between Faecalibacterium abundance and depression severity; - Modulators of gut microbiota (antibiotics, probiotics and FMT) were experienced only in experimental settings in ASD/MDs with promising results
Colpo et al., 2017 [101]	Review	-	Selected studies on the role of inflammation and immune-based therapeutic strategies in MDs	- Treatment with probiotics may improve behavioural symptoms (Decreased depression-like and anxiety-like behaviours) by acting on monoaminergic systems (e.g., increased serotonin availability) and/or decreasing levels of systemic inflammatory markers (decreased IL-1β, IL-6, TNF-α, microglial activation markers) in animal models and improve anxious and depressive symptoms in humans
Jiang et al., 2015 [106]	Cross-sectional study	46 depressed patients (active MDD and responded MDD) and 30 HC	Comparing blood samples and faecal samples using high-throughput pyrosequencing	- Increased faecal bacterial alpha-diversity in the active-MDD, but not in the responded-MDD, compared to the HC group; differences in the composition of microbiota between groups (Increased Bacteroidetes, Proteobacteria, Actinobacteria, Enterobacteriaceae and Alistipes decreased Firmicutes, and Faecalibacterium in MDD patients); negative correlation between Faecalibacterium and severity of depressive symptoms; - No difference in the serum inflammatory markers, while the serum level of BDNF differed significantly between the groups
Aizawa et al., 2016 [107]	Cross-sectional study	43 MDD patients and 57 HC	Comparing faecal samples using bacterial rRNA-targeted reverse transcription-quantitative PCR	- Decreased Bifidobacterium and/or Lactobacillus in patients compared to controls
Zheng et al., 2016 [108]	Cross-sectional study; animal study (mice)	GF and SPF Kunming mice	Open-field test, Y-maze, tail suspension test, forced swimming test; 16S rRNA gene sequencing on faecal samples from MDD patients and HC; FMT	- Depression-like behaviours in GF mice (decreased immobility time in the forced swimming test); - Significant differences in microbiota composition of MDD patients and HC; - Depression-like behaviours and disturbances of microbial genes and host metabolites involved in carbohydrate and amino acid metabolism in GF mice after transplantation with faecal samples from MDD patients
Evans et al., 2017 [109]	Cross-sectional study	115 BD patients and 64 HC	Comparing faecal samples using 16S rRNA gene sequence analysis; psychometric evaluations	- Decrease Faecalibacterium in BD; - Significant relationships between the fractional representation of several operational taxonomical units and the self-reported burden of disease measures within BD individuals
Flowers et al., 2017 [110]	Cross-sectional study	117 BD patients (AAP-treated or non-AAP-treated)	Comparing faecal samples using 16S ribosomal sequencing	- Decreased species diversity in AAP-treated females; - Differences in the composition of microbiota between treatment groups (Lachnospiraceae, Akkermansia and Sutterella)
Painold et al., 2019 [112]	Cross-sectional study	32 BD patients and 10 HC	Comparing blood samples and faecal samples using 16S rRNA gene sequencing	- Negative correlation between microbial alpha-diversity and illness duration; - Increased Actinobacteria and Coriobacteria in BD, increased Ruminococcaceae and Faecalibacterium in HC; - Increased Lactobacillales, Streptococcaceae and Bacilli in BD individuals with higher IL-6 levels; Increased Faecalibacterium in BD individuals with higher malondialdehyde levels; tryptophan levels associated with the family of Lactobacillaceae
Huang et al., 2019 [113]	Review	-	12 selected human studies	- Decreased microbial diversity in depressed patients (Increased Actinobacteria, Enterobacteriaceae and decreased Faecalibacterium); - Specific gut bacteria were associated with inflammatory markers and metabolic profiles, disease severity, duration of illness, psychiatric symptoms and pharmacological treatment
Maes et al., 2008 [114]	Cross-sectional study	MDD patients and HC	Comparing blood samples	- Increased serum IgM and IgA against LPS of enterobacteria in MDD patients
Slyepchenko et al., 2017 [115]	Narrative review	-	2016 selected studies on the role of intestinal dysbiosis in the pathophysiology of MDD and somatic comorbidities	- Gut dysbiosis and the leaky gut may influence several pathways implicated in the biology of MDD and related medical comorbidities
Kelly et al., 2016 [116]	Cross-sectional study; animal study (rats)	34 MDD patients and 33 matched HC	Comparing blood, salivary and faecal samples; FMT to a microbiota-deficient rat model	- Decreased gut microbiota richness and diversity in MDD patients (decreased Prevotellaceae) and rats after FMT from MDD patients; - Behavioural (anhedonia, anxiety-like behaviours) and physiological depressive features (increased CRP and intestinal transit time) and alterations in tryptophan metabolism (increased kynurenine/tryptophan ratio) in mice after faecal transplantation from MDD patients
Yang et al., 2020 [117]	Cross-sectional study	156 MDD patients and 155 HC	Whole-genome shotgun metagenomic and untargeted metabolomic methods	- Increassed Bacteroides, decreased Blautia and Eubacterium in MDD patients
Patterson et al., 2019 [122]	Animal study (mice)	Diet-induced obese and metabolically dysfunctional mice	Daily administration of GABA-producing L. brevis (L. brevis DPC6108 or L. brevis DSM32386) for 12 weeks	- Decreased accumulation of mesenteric adipose tissue, increassed insulin secretion following glucose challenge and plasma cholesterol clearance; - Decreassed despair-like behaviour and basal corticosterone production during the forced swim test
Naseribafrouei et al., 2014 [125]	Cross-sectional study	37 depressed patients and 18 HC	Comparing faecal samples using 16S rRNA gene sequencing	- Increassed Bacteroidales and decreased Lachnospiraceae in depressed patients; - Significant association between depression and one clade within the genus Oscillibacter and one clade within Alistipes
Severance et al., 2016 [127]	Cross-sectional study	Two cohorts totaling 947 individuals with SZ and BD, as well as HC	Comparing blood samples in patients with SZ and BD, as well as HC	- C. albicans seropositivity increased the odds for an SZ diagnosis in males, decreased cognitive scores in SZ females and correlated with decreased performance on memory modules for both disorders; - C. albicans IgG levels were not impacted by antipsychotic medication; - Elevated C. albicans levels in males with SZ and females with BD were associated with GI disturbances
Dickerson et al., 2017 [128]	Review	-	Selected human studies on the relationship between immune alterations and microbiome in SZ and BD	- Microbiome may affect cognition and behaviour by altering the functioning of the immune system (animal studies); - Evidence of increased gastrointestinal inflammation in SZ and BD based on measures of microbial translocation; - Increassed rate of recent antimicrobial prescription in patients with acute mania, which were associated with Increassed severity of mania symptoms
Macedo et al., 2017 [129]	Narrative review	-	120 selected articles on the mutual relationship between stress, depression and gut microbiota composition and antimicrobial effect of ADs and vice versa	- MDD was associated with changes in gut permeability and microbiota composition; - ADs presented antimicrobial effects and, conversely, some antimicrobials presented antidepressant effects

Legend: AAP—atypical antipsychotics; Ads—antidepressants; ASD—autism spectrum disorders; BD—bipolar disorder; BDNF—brain-derived neurotrophic factor; C. albicans—Candida albicans; CRP—C reactive protein; FMT—faecal microbiota transplantation; GABA—gamma-aminobutyric acid; GF—germ-free; GI—gastrointestinal; HC—healthy controls; L. brevis—Lactobacillus brevis; LPS—lipopolysaccharide; MDD—major depressive disorder; PCR—polymerase chain reaction; SPF—specific pathogen-free; SZ—schizophrenia; TNF-α—tumor necrosis factor alpha.

**Table 2:** Studies on the relationships between microbiota and obsessive-compulsive disorder (OCD).

Authors and Year	Type of Study	Findings
Kantak et al., 2014 [132]	Animal study (BALB/cJ house mice)	- Pretreatment with probiotics (L. rhamnosus GG) or with fluoxetine attenuated the OCD-like behaviours induced by RU 24969 in comparison with saline pretreatment; - The effects of L. rhamnosus pretreatment and fluoxetine pretreatment on OCD-like behaviours were comparable
Sanikhani et al., 2020 [135]	Animal study (rats)	- Treatment with L. casei Shirota, with fluoxetine and with the combination of both reduced OCD-like symptoms induced by quinpirole hydrochloride; - L. casei shirota might modulate gene expression (Increassed BDNF, decreassed 5-HT2A receptors) in the OFC
Kobliner et al., 2018 [136]	Case report	S. boulardii administration, aimed at reducing GI symptoms, resulted in an amelioration of OCD and SIB in a boy with ASD, OCD, tics, SIB, a history of GI disturbances and global immune dysregulation
Rees et al., 2014 [140]	Review	Antibiotics altering the composition of intestinal flora could be the causative factor of PANDAS rather than GABHS

Legend: BDNF—brain-derived neurotrophic factor; GABHS—group A beta-haemolytic streptococcus; GI—gastrointestinal; L. casei Shirota—Lactobacillus casei Shirota; L. rhamnosus GG—Lactobacillus rhamnosus GG; OFC—orbitofrontal cortex; PANDAS—paediatric autoimmune neuropsychiatric disorders associated with streptococcal infections; S. boulardii—Saccharomyces boulardii; SIB—self-injurious behaviour.

**Table 3:** Studies on the relationships between gut microbiota and schizophrenia (SZ).

Authors and Year	Type of Study	Methods	Findings
Zheng et al., 2019 [160]	Cross-sectional study; animal study	Comparing gut microbiota between 63 treated and untreated SZ patients and 69 HCs; GF mice received SZ FMT	- Both treated and untreated SZ subjects showed altered microbiota and decreased microbiome heterogeneity than HC; - SZ severity was correlated with unique bacterial taxa; - GF mice receiving SZ FMT showed lower glutamate and higher glutamine and GABA in the hippocampus and displayed SZ-relevant behaviours
Shen et al., 2018 [161]	Cross-sectional study	Comparing gut microbiota between 64 SZ patients and 53 HC using 16S rRNA sequencing	- Increassed Proteobacteria, Succinivibrio, Megasphaera, Collinsella, Clostridium, Klebsiella and Methanobrevibacter in SZ; - Decreassed Blautia, Coprococcus and Roseburia in SZ; - Proposed microbiota-based diagnosis for SZ
Yuan et al., 2018 [162]	Cross-sectional study	Comparing gut microbiota between 41 first-episode SZ patients and 41 HCs; testing 24-week risperidone treatment effects	- Altered microbiota composition in patients, modulated by risperidone treatment
Li et al., 2021 [171]	Cross-sectional study	Investigating faecal microbiota differences between 38 SZ patients and 38 HC, as well as exploring whether such differences were associated with brain structure and function, through 16S rRNA sequencing, sMRI and rs-fMRI	- SZ showed increassed Veillonella, decreased Ruminococcus, Roseburia, GMV and ReHo; increased amplitudes of low-frequency fluctuation, - Both GMV and ReHo were related to the diversity of gut microbiota

Legend: GABA—gamma-aminobutyric acid; GF—germ-free; GMV—gray matter volume; HC—healthy controls; FMT—faecal microbiota transplant; ReHo—regional homogeneity; rs-fMRI—resting-state functional magnetic resonance imaging; sMRI—structural magnetic resonance imaging.

**Table 4:** Studies on gastrointestinal (GI) symptoms and gut microbiota composition in autism spectrum disorder (ASD).

Authors and Year	Type of Study	Participants (N)	Methods	Findings
McElhanon et al., 2014 [181]	Systematic meta-analysis	ASD group: 2215; comparison group: 50664	15 studies included in the systematic review	Greater prevalence of GI symptoms among children with ASD compared with control children
Adams et al., 2011 [182]	Cross-sectional study	58 ASD children; 39 healthy controls	GI symptoms: assessed with a modified six-item GI Severity Index (6-GSI) questionnaire; autistic symptoms: assessed with the Autism Treatment Evaluation Checklist (ATEC)	Correlations between GI symptoms and autism severity
Kang et al., 2017 [183]	Open-label trial	18 ASD-diagnosed children	MTT	- Decreased GI symptoms; - Improvement of ASD symptoms; - Changes in microbiota composition
Gondalia et al., 2012 [196]	Cross-sectional study	28 autistic children with GI dysfunction; 23 autistic children without GI dysfunction; 53 neurotypical siblings	Comparing gut microbiota	No significant difference between groups
Sandler et al., 2000 [201]	Open-label trial	11 children with regressive-onset autism	Administration of vancomycin	Short-term behavioural improvement

Legend: MTT—microbiota transfer therapy.

**Table 5:** Studies on the relationships between gut microbiota and attention-deficit/hyperactivity disorder (ADHD).

Authors and Year	Type of Study	Findings
Cenit et al., 2017 [89]	Review	- Gut microbiota transplantation can transfer a behavioural phenotype (studies on animal samples); - Microbiota could play a role in mental health by regulating inflammatory and endocrine secretions, synthetising neuroactive compounds and interacting with the vagal nerve (studies on animal samples); - Dietary components modulating gut microbiota may influence ADHD development or symptoms (preliminary human studies)
Cenit et al., 2017 [202]	Review	- Specific diets or dietary components (including probiotics) may alter brain activity in regions that are relevant to cognition, behaviour and specific ADHD symptoms; - Inflammation and oxidative stress, partly triggered by alterations in gut microbiota composition and both associated with ADHD, may play an important role in the aetiopathogenesis of ADHD through neuroinflammation

**Table 6:** Studies on the relationships between gut microbiota and eating disorders.

Authors and Year	Type of Study	Findings
Santonicola et al., 2019 [203]	Review	- Differences in alpha-diversity and composition of microbiota in EDs, possibly contributing to symptomatic manifestations and pathophysiology;
Seitz et al., 2019 [204]	Review	- Decreased alpha-diversity in AN, which showed an increase during weight restoration and a correlation with depressive and anxious symptoms; - Increased beta-diversity in AN, which decreased after weight rehabilitation; - Specific taxa abundance in AN could influence gut permeability, inflammation and symptomatic manifestations
Seitz et al., 2019 [205]	Review	- Decreased diversity and taxa abundance in AN; - AN-related changes in microbiome could increase gut permeability, inflammation and autoantibody formation; - Increased microbiome diversity in AN associated with depressive, anxious and EDs symptoms

Legend: AN—anorexia nervosa, ED—eating disorders.

## Data Availability

All data generated or analysed during this study are included in this published article.

## References

[1] Gill S.R., Pop M., Deboy R.T., Eckburg P.B., Turnbaugh P.J., Samuel B.S., Gordon J.I., Relman D.A., Fraser-Liggett C.M., Nelson K.E. (2006). Metagenomic analysis of the human distal gut microbiome. Science.

[2] Clemente J.C., Ursell L.K., Parfrey L.W., Knight R. (2012). The impact of the gut microbiota on human health: An integrative view. Cell.

[3] Ma Q., Xing C., Long W., Wang H.Y., Liu Q., Wang R.F. (2019). Impact of microbiota on central nervous system and neurological diseases: The gut-brain axis. J. Neuroinflamm..

[4] Stecher B., Hardt W.D. (2011). Mechanisms controlling pathogen colonization of the gut. Curr. Opin. Microbiol..

[5] Tilg H., Kaser A. (2011). Gut microbiome, obesity, and metabolic dysfunction. J. Clin. Investig..

[6] Collins S.M., Surette M.G., Bercik P. (2012). The interplay between the intestinal microbiota and the brain. Nat. Rev. Microbiol..

[7] Ojeda J., Ávila A., Vidal P.M. (2021). Gut microbiota interaction with the central nervous system throughout life. J. Clin. Med..

[8] Rinninella E., Cintoni M., Raoul P., Lopetuso L.R., Scaldaferri F., Pulcini G., Miggiano G., Gasbarrini A., Mele M.C. (2019). Food components and dietary habits: Keys for a healthy gut microbiota composition. Nutrients.

[9] Dinan T.G., Cryan J.F. (2012). Regulation of the stress response by the gut microbiota: Implications for psychoneuroendocrinology. Psychoneuroendocrinology.

[10] Redondo-Useros N., Nova E., González-Zancada N., Díaz L.E., Gómez-Martínez S., Marcos A. (2020). Microbiota and lifestyle: A special focus on diet. Nutrients.

[11] Borre Y.E., O’Keeffe G.W., Clarke G., Stanton C., Dinan T.G., Cryan J.F. (2014). Microbiota and neurodevelopmental windows: Implications for brain disorders. Trends. Mol. Med..

[12] Chu D.M., Antony K., Ma J., Prince A.L., Showalter L., Moller M., Aagaard K.M. (2016). The early infant gut microbiome varies in association with a maternal high-fat diet. Genome Med..

[13] Chu D.M., Ma J., Prince A.L., Antony K.M., Seferovic M.D., Aagard K.M. (2017). Maturation of the infant microbiome community structure and function across multiple body sites and in relation to mode of delivery. Nat. Med..

[14] Furness J.B. (2012). The enteric nervous system and neurogastroenterology. Nat. Rev. Gastroenterol. Hepatol..

[15] Mayer E.A. (2011). Gut feelings: The emerging biology of gut-brain communication. Nat. Rev. Neurosci..

[16] Carabotti M., Scirocco A., Maselli M.A., Severi C. (2015). The gut-brain axis: Interactions between enteric microbiota, central and enteric nervous systems. Ann. Gastroenterol..

[17] Ono S., Karaki S.I., Kuwahara A. (2004). Short-chain fatty acids decrease the frequency of spontaneous contractions of longitudinal muscle via enteric nerves in rat distal colon. Jpn. J. Physiol..

[18] Morita C., Tsuji H., Hata T., Gondo M., Takakura S., Kawai K., Yoshihara K., Ogata K., Nomoto K., Miyazaki K. (2015). Gut dysbiosis in patients with anorexia nervosa. PLoS ONE.

[19] Benakis C., Brea D., Caballero S., Faraco G., Moore J., Murphy M., Sita G., Racchumi G., Ling L., Pamer E.G. (2016). Commensal microbiota affects ischemic stroke outcome by regulating intestinal γδ T cells. Nat. Med..

[20] Sampson T.R., Debelius J.W., Thron T., Janssen S., Shastri G.G., Ilhan Z.E., Challis C., Schretter C.E., Rocha S., Gradinaru V. (2016). Gut microbiota regulate motor deficits and neuroinflammation in a model of Parkinson’s disease. Cell.

[21] Sharon G., Sampson T.R., Geschwind D.H., Mazmanian S.K. (2016). The central nervous system and the gut microbiome. Cell.

[22] Olson C.A., Vuong H.E., Yano J.M., Liang Q.Y., Nusbaum D.J., Hsiao E.Y. (2018). The gut microbiota mediates the anti-seizure effects of the ketogenic diet. Cell.

[23] Bojović K., Ignjatović Ð.I., Soković Bajić S., Vojnović Milutinović D., Tomić M., Golić N., Tolinački M. (2020). Gut microbiota dysbiosis associated with altered production of short chain fatty acids in children with neurodevelopmental disorders. Front. Cell. Infect. Microbiol..

[24] Halverson T., Alagiakrishnan K. (2020). Gut microbes in neurocognitive and mental health disorders. Ann. Med..

[25] Wu M., Tian T., Mao Q., Zou T., Zhou C.J., Xie J., Chen J.J. (2020). Associations between disordered gut microbiota and changes of neurotransmitters and short-chain fatty acids in depressed mice. Transl. Psychiatry.

[26] Masand P.S., Keuthen N.J., Gupta S., Virk S., Yu-Siao B., Kaplan D. (2006). Prevalence of irritable bowel syndrome in obsessive–compulsive disorder. CNS Spectr..

[27] Wu J.C. (2012). Psychological co-morbidity in functional gastrointestinal disorders: Epidemiology, mechanisms and management. J. Neurogastroenterol. Motil..

[28] Sibelli A., Chalder T., Everitt H., Workman P., Windgassen S., Moss-Morris R. (2016). A systematic review with meta-analysis of the role of anxiety and depression in irritable bowel syndrome onset. Psychol. Med..

[29] Turna J., Kaplan K.G., Patterson B., Bercik P., Anglin R., Soreni N., Van Ameringen M. (2019). Higher prevalence of irritable bowel syndrome and greater gastrointestinal symptoms in obsessive-compulsive disorder. J. Psychiatr. Res..

[30] Reyes R.E.N., Zhang Z., Gao L., Asatryan L. (2020). Microbiome meets microglia in neuroinflammation and neurological disorders. Neuroimmunol. Neuroinflamm..

[31] Houser M.C., Tansey M.G. (2017). The gut-brain axis: Is intestinal inflammation a silent driver of Parkinson’s disease pathogenesis?. NPJ Parkinson’s Dis..

[32] Warner B.B. (2019). The contribution of the gut microbiome to neurodevelopment and neuropsychiatric disorders. Pediatr. Res..

[33] Zhu S., Jiang Y., Xu K., Cui M., Ye W., Zhao G., Jin L., Chen X. (2020). The progress of gut microbiome research related to brain disorders. J. Neuroinflamm..

[34] Mehrian-Shai R., Reichardt J.K.V., Harris C.C., Toren A. (2019). The gut-brain axis, paving the way to brain cancer. Trends Cancer.

[35] Phillips M.L., Swartz H.A. (2014). A critical appraisal of neuroimaging studies of bipolar disorder: Toward a new conceptualization of underlying neural circuitry and a road map for future research. Am. J. Psychiatry.

[36] Zhou Y., Fan L., Qiu C., Jiang T. (2015). Prefrontal cortex and the dysconnectivity hypothesis of schizophrenia. Neurosci. Bull..

[37] Karim H.T., Wang M., Andreescu C., Tudorascu D., Butters M.A., Karp J.F., Reynolds C.F., Aizenstein H.J. (2018). Acute trajectories of neural activation predict remission to pharmacotherapy in late-life depression. Neuroimage Clin..

[38] Liang S., Wu X., Jin F. (2018). Gut-brain psychology: Rethinking psychology from the microbiota-gut-brain axis. Front. Integr. Neurosci..

[39] Moher D., Liberati A., Tetzlaff J., Altman D.G., PRISMA Group (2009). Preferred reporting items for systematic reviews and meta-analyses: The PRISMA statement. PLoS Med..

[40] Cryan J.F., Dinan T.G. (2012). Mind-altering microorganisms: The impact of the gut microbiota on brain and behaviour. Nat. Rev. Neurosci..

[41] Foster J.A., McVey Neufeld K.A. (2013). Gut-brain axis: How the microbiome influences anxiety and depression. Trends Neurosci..

[42] Turna J., Grosman Kaplan K., Anglin R., Van Ameringen M. (2016). “What’s bugging the gut in ocd?” A review of the gut microbiome in obsessive-compulsive disorder. Depress. Anxiety.

[43] Quigley E.M.M. (2017). Microbiota-brain-gut axis and neurodegenerative diseases. Curr. Neurol. Neurosci. Rep..

[44] Heijtz R.D., Wang S., Anuar F., Qian Y., Bjorkholm B., Samuelsson A., Hibberd M.L., Forssberg H., Pettersson S. (2011). Normal gut microbiota modulates brain development and behavior. Proc. Natl. Acad. Sci. USA.

[45] Neufeld K.A., Kang N., Bienenstock J., Foster J.A. (2011). Effects of intestinal microbiota on anxiety-like behavior. Commun. Integr. Biol..

[46] Clarke G., Grenham S., Scully P., Fitzgerald P., Moloney R.D., Shanahan F., Dinan T.G., Cryan J.F. (2013). The microbiome-gut-brain axis during early life regulates the hippocampal serotonergic system in a sex-dependent manner. Mol. Psychiatry.

[47] Luczynski P., Whelan S.O., O’Sullivan C., Clarke G., Shanahan F., Dinan T.G., Cryan J.F. (2016). Adult microbiota-deficient mice have distinct dendritic morphological changes: Differential effects in the amygdala and hippocampus. Eur. J. Neurosci..

[48] Lu J., Lu L., Yu Y., Cluette-Brown J., Martin M.C., Claud C.E. (2018). Effects of intestinal microbiota on brain development in humanized gnotobiotic mice. Sci. Rep..

[49] Hoban A.E., Stilling R.M., Ryan F.J., Shanahan F., Dinan T.G., Claesson M.J., Clarke G., Cryan J.F. (2016). Regulation of prefrontal cortex myelination by the microbiota. Transl. Psychiatry.

[50] Desbonnet L., Garrett L., Clarke G., Bienenstock J., Dinan T.G. (2008). The probiotic Bifidobacteria infantis: An assessment of potential antidepressant properties in the rat. J. Psychiatr. Res..

[51] Bravo J.A., Forsythe P., Chew M.V., Escaravage E., Savignac H.M., Dinan T.G., Bienenstock J., Cryan J.F. (2011). Ingestion of Lactobacillus strain regulates emotional behavior and central GABA receptor expression in a mouse via the vagus nerve. Proc. Natl. Acad. Sci. USA.

[52] Barrett E., Ross R.P., O’Toole P.W., Fitzgerald G.F., Stanton C. (2012). γ-Aminobutyric acid production by culturable bacteria from the human intestine. J. Appl. Microbiol..

[53] Amireault P., Sibon D., Cote F. (2013). Life without peripheral serotonin: Insights from tryptophan hydroxylase 1 knockout mice reveal the existence of paracrine/autocrine serotonergic networks. ACS Chem. Neurosci..

[54] Yano J.M., Yu K., Donaldson G.P., Shastri G.G., Ann P., Ma L., Nagler C.R., Ismagilov R.F., Mazmanian S.K., Hsiao E.Y. (2015). Indigenous bacteria from the gut microbiota regulate host serotonin biosynthesis. Cell.

[55] Rudzki L., Szulc A. (2018). “Immune gate” of psychopathology—The role of gut derived immune activation in major psychiatric disorders. Front. Psychiatry.

[56] O’Mahony S.M., Marchesi J.R., Scully P., Codling C., Ceolho A.M., Quigley E.M., Cryan J.F., Dinan T.G. (2009). Early life stress alters behavior, immunity, and microbiota in rats: Implications for irritable bowel syndrome and psychiatric illnesses. Biol. Psychiatry.

[57] Bailey M.T., Dowd S.E., Galley J.D., Hufnagle A.R., Allen R.G., Lyte M. (2011). Exposure to a social stressor alters the structure of the intestinal microbiota: Implications for stressor-induced immunomodulation. Brain Behav. Immun..

[58] Bangsgaard Bendtsen K.M., Krych L., Sørensen D.B., Pang W., Nielsen D.S., Josefsen K., Hansen L.H., Sørensen S.J., Hansen A.K. (2012). Gut microbiota composition is correlated to grid floor induced stress and behavior in the BALB/c mouse. PLoS ONE.

[59] Brenner D.J., Fanning G.R., Johnson K.E., Citarella R.V., Falkow S. (1969). Polynucleotide sequence relationships among members of Enterobacteriaceae. J. Bacteriol..

[60] Campisi J., Leem T.H., Fleshner M. (2003). Stress-induced extracellular Hsp72 is a functionally significant danger signal to the immune system. Cell. Stress Chaperones..

[61] Kluge M., Schüssler P., Künzel H.E., Dresler M., Yassouridis A., Steiger A. (2007). Increased nocturnal secretion of ACTH and cortisol in obsessive compulsive disorder. J. Psychiatr. Res..

[62] Zimomra Z.R., Porterfield V.M., Camp R.M., Johnson J.D. (2011). Time-dependent mediators of HPA axis activation following live Escherichia coli. Am. J. Physiol. Regul. Integr. Comp. Physiol..

[63] Gehrmann J., Banati R.B., Kreutzberg G.W. (1993). Microglia in the immune surveillance of the brain: Human microglia constitutively express HLA-DR molecules. J. Neuroimmunol..

[64] Erny D., Hrabě de Angelis A.L., Jaitin D., Wieghofer P., Staszewski O., David E., Keren-Shaul H., Mahlakoiv T., Jakobshagen K., Buch T. (2015). Host microbiota constantly control maturation and function of microglia in the CNS. Nat. Neurosci..

[65] Priller J., Prinz M. (2019). Targeting microglia in brain disorders. Science.

[66] Fazekas de St Groth B. (2012). Regulatory T-cell abnormalities and the global epidemic of immuno-inflammatory disease. Immunol. Cell. Biol..

[67] Marazziti D., Hollander E., Lensi P., Ravagli S., Cassano G.B. (1992). Peripheral markers of serotonin and dopamine function in obsessive-compulsive disorder. Psychiatry Res..

[68] Benros M.E., Eaton W.W., Mortensen P.B. (2014). The epidemiologic evidence linking autoimmune diseases and psychosis. Biol. Psychiatry.

[69] Al-Diwani A.A.J., Pollak T.A., Irani S.R., Lennox B.R. (2017). Psychosis: An autoimmune disease?. Immunology.

[70] Gerentes M., Pelissolo A., Rajagopal K., Tamouza R., Hamdani N. (2019). Obsessive-compulsive disorder: Autoimmunity and neuroinflammation. Curr. Psychiatry Rep..

[71] Jeppesen R., Benros M.E. (2019). Autoimmune diseases and psychotic disorders. Front. Psychiatry.

[72] Marazziti D., Mucci F., Fontanelle L.F. (2018). Immune system and obsessive-compulsive disorder. Psychoneuroendocrinology.

[73] Rogers J.P., Pollak T.A., Blackman G., David A.S. (2019). Catatonia and the immune system: A review. Lancet Psychiatry.

[74] Bischoff S.C., Barbara G., Buurman W., Ockhuizen T., Schulzke J.D., Serino M., Tilg H., Watson A., Wells J.M. (2014). Intestinal permeability–A new target for disease prevention and therapy. BMC Gastroenterol..

[75] Kelly J.R., Kennedy P.J., Cryan J.F., Dinan T.G., Clarke G., Hyland N.P. (2015). Breaking down the barriers: The gut microbiome, intestinal permeability and stress-related psychiatric disorders. Front. Cell. Neurosci..

[76] Tetz G., Tetz V. (2016). Bacteriophage infections of microbiota can lead to leaky gut in an experimental rodent model. Gut Pathog..

[77] Yarandi S.S., Peterson D.A., Treisman G.J., Moran T.H., Pasricha P.J. (2016). Modulatory effects of gut microbiota on the central nervous system: How gut could play a role in neuropsychiatric health and diseases. J. Neurogastroenterol. Motil..

[78] Braniste V., Al-Asmakh M., Kowal C., Anuar F., Abbaspour A., Tóth M., Korecka A., Bakocevic N., Ng L.G., Kundu P. (2014). The gut microbiota influences blood-brain barrier permeability in mice. Sci. Transl. Med..

[79] Fröhlich E.E., Farzi A., Mayerhofer R., Reichmann F., Jačan A., Wagner B., Zinser E., Bordag N., Magnes C., Fröhlich E. (2016). Impairment by antibiotic-induced gut dysbiosis: Analysis of gut microbiota-brain communication. Brain Behav. Immun..

[80] Obrenovich M.E.M. (2018). Leaky gut, leaky brain?. Microorganisms.

[81] Dinan T.G., Stanton C., Cryan J.F. (2013). Psychobiotics: A novel class of psychotropic. Biol. Psychiatry.

[82] Grossi E., Terruzzi V. (2014). The role of intestinal dysbiosis in the pathogenesis of autism: Minireview. Int. J. Microbiol. Adv. Immunol..

[83] Reddy B.L., Saier M.H. (2015). Autism and our intestinal microbiota. J. Mol. Microbiol. Biotechnol..

[84] Mangiola F., Ianiro G., Franceschi F., Fagiuoli S., Gasbarrini G., Gasbarrini A. (2016). Gut microbiota in autism and mood disorders. World J. Gastroenterol..

[85] Sarkar A., Lehto S.M., Harty S., Dinan T.G., Cryan J.F., Burnet P.W.J. (2016). Psychobiotics and the manipulation of bacteria-gut-brain signals. Trends Neurosci..

[86] Wang H.X., Wang Y.P. (2016). Gut microbiota-brain axis. Chin. Med. J..

[87] Kelly J.R., Minuto C., Cryan J.F., Clarke G., Dinan T.G. (2017). Cross talk: The microbiota and neurodevelopmental disorders. Front. Neurosci..

[88] Parker A., Fonseca S., Carding S.R. (2020). Gut microbes and metabolites as modulators of blood-brain barrier integrity and brain health. Gut Microbes.

[89] Cenit M.C., Sanz Y., Codoñer-Franch P. (2017). Influence of gut microbiota on neuropsychiatric disorders. World J. Gastroenterol..

[90] American Psychiatric Association (2013). Diagnostic and Statistical Manual of Mental Disorders.

[91] Akiskal H.S. (1986). A developmental perspective on recurrent mood disorders: A review of studies in man. Psychopharmacol. Bull..

[92] Engel C. (2008). Mood Disorders in Adolescent medicine.

[93] Krishnan V., Nestler E.J. (2010). Linking molecules to mood: New insight into the biology of depression. Am. J. Psychiatry.

[94] Pitchot W., Scantamburlo G., Ansseau M., Souery D. (2012). Bipolar disorder: A multifactorial disease. Rev. Med. Liege.

[95] Price J.L., Drevets W.C. (2012). Neural circuits underlying the pathophysiology of mood disorders. Trends Cogn. Sci..

[96] Gonda X., Petschner P., Eszlari N., Baksa E., Edes A., Antal P., Juhasz G., Bagdy G. (2019). Genetic variants in major depressive disorder: From pathophysiology to therapy. Pharmacol. Ther..

[97] Rowland T.A., Marwaha S. (2018). Epidemiology and risk factors for bipolar disorder. Ther. Adv. Psychopharmacol..

[98] McNamara R.K., Lotrich F.E. (2012). Elevated immune-inflammatory signaling in mood disorders: A new therapeutic target?. Expert Rev. Neurother..

[99] Rosenblat J.D., Cha D.S., Mansur R.B., McIntyre R.S. (2014). Inflamed moods: A review of the interactions between inflammation and mood disorders. Prog. Neuropsychopharmacol. Biol. Psychiatry.

[100] Bhattacharya A., Derecki N.C., Lovenberg T.W., Drevets W.C. (2016). Role of neuro-immunological factors in the pathophysiology of mood disorders. Psychopharmacology.

[101] Colpo G.D., Leboyer M., Dantzer R., Trivedi M.H., Teixeira A.L. (2018). Immune-based strategies for mood disorders: Facts and challenges. Expert Rev. Neurother..

[102] Mucci F., Marazziti D., Della Vecchia A., Baroni S., Morana P., Carpita B., Mangiapane P., Morana F., Morana B., Dell’Osso L. (2020). State-of-the-Art: Inflammatory and metabolic markers in mood disorders. Life.

[103] Sotelo J.L., Nemeroff C.B. (2017). Depression as a systemic disease. Personalized Med. Psychiatry.

[104] Sousa S. (2020). Depression as a Systemic Illness Edited by James J. Strain, Michael Blumenfield Oxford University Press. 2018. 336 pp. ISBN 9780190603342. Br. J. Psychiatry.

[105] Strandwitz P. (2018). Neurotransmitter modulation by the gut microbiota. Brain Res..

[106] Jiang H., Ling Z., Zhang Y., Mao H., Ma Z., Yin Y., Wang W., Tang W., Tan Z., Shi J. (2015). Altered fecal microbiota composition in patients with major depressive disorder. Brain Behav. Immun..

[107] Aizawa E., Tsuji H., Asahara T., Takahashi T., Teraishi T., Yoshida S., Ota M., Koga N., Hattori K., Kunugi K. (2016). Possible association of Bifidobacterium and Lactobacillus in the gut microbiota of patients with major depressive disorder. J. Affect. Disord..

[108] Zheng P., Zeng B., Zhou C., Liu M., Fang Z., Xu X., Zeng L., Chen J., Fan S., Du X. (2016). Gut microbiome remodeling induces depressive-like behaviors through a pathway mediated by the host’s metabolism. Mol. Psychiatry.

[109] Evans S.J., Bassis C.M., Hein R., Assari S., Flowers S.A., Kelly M.B., Young V.B., Ellingrod V.E., McInnis M.G. (2017). The gut microbiome composition associates with bipolar disorder and illness severity. J. Psychiatr. Res..

[110] Flowers S.A., Evans S.J., Ward K.M., McInnis M.G., Ellingrod V.E. (2017). Interaction between atypical antipsychotics and the gut microbiome in a bipolar disease cohort. Pharmacotherapy.

[111] Lin L., Zhang J. (2017). Role of intestinal microbiota and metabolites on gut homeostasis and human diseases. BMC Immunol..

[112] Painold A., Mörkl S., Kashofer K., Halwachs B., Dalkner N., Bengesser S., Birner A., Fellendorf F., Platzer M., Queissner R. (2019). A step ahead: Exploring the gut microbiota in inpatients with bipolar disorder during a depressive episode. Bipolar Disord..

[113] Huang T.T., Lai J.B., Du Y.L., Xu Y., Ruan L.M., Hu S.H. (2019). Current understanding of gut microbiota in mood disorders: An update of human studies. Front. Genet..

[114] Maes M., Kubera M., Leunis J.C. (2008). The gut-brain barrier in major depression: Intestinal mucosal dysfunction with an increased translocation of LPS from gram negative enterobacteria (leaky gut) plays a role in the inflammatory pathophysiology of depression. Neuro Endocrinol. Lett..

[115] Slyepchenko A., Maes M., Jacka F.N., Köhler C.A., Barichello T., McIntyre R.S., Berk M., Grande I., Foster J.A., Vieta E. (2017). Gut microbiota, bacterial translocation, and interactions with diet: Pathophysiological links between major depressive disorder and non-communicable medical comorbidities. Psychother. Psychosom..

[116] Kelly J.R., Borre Y., O’Brien C., Patterson E., El Aidy S., Deane J., Kennedy P.J., Beers S., Scott K., Moloney G. (2016). Transferring the blues: Depression-associated gut microbiota induces neurobehavioural changes in the rat. J. Psychiatr. Res..

[117] Yang J., Zheng P., Li Y., Wu J., Tan X., Zhou J., Sun Z., Chen X., Zhang G., Zhang H. (2020). Landscapes of bacterial and metabolic signatures and their interaction in major depressive disorders. Sci. Adv..

[118] Maier E., Anderson R.C., Roy N.C. (2015). Understanding how commensal obligate anaerobic bacteria regulate immune functions in the large intestine. Nutrients.

[119] Schiepers O.J., Wichers M.C., Maes M. (2005). Cytokines and major depression. Prog. Neuropsychopharmacol. Biol. Psychiatry.

[120] Bajaj J.S., Hylemon P.B., Ridlon J.M., Heuman D.M., Daita K., White M.B., Monteith P., Noble N.A., Sikaroodi M., Gillevet P.M. (2012). Colonic mucosal microbiome differs from stool microbiome in cirrhosis and hepatic encephalopathy and is linked to cognition and inflammation. Am. J. Physiol. Gastrointest. Liver Physiol..

[121] Pan J.X., Xia J.J., Deng F.L., Liang W.W., Wu J., Yin B.M., Dong M.X., Chen J.J., Ye F., Wang H.Y. (2018). Diagnosis of major depressive disorder based on changes in multiple plasma neurotransmitters: A targeted metabolomics study. Transl. Psychiatry.

[122] Patterson E., Ryan P.M., Wiley N., Carafa I., Sherwin E., Moloney G., Franciosi E., Mandal R., Wishart D.S., Tuohy K. (2019). Gamma-aminobutyric acid-producing lactobacilli positively affect metabolism and depressive-like behaviour in a mouse model of metabolic syndrome. Sci. Rep..

[123] Prevot T., Sibille E. (2020). Altered GABA-mediated information processing and cognitive dysfunctions in depression and other brain disorders. Mol. Psychiatry..

[124] Frémont M., Coomans D., Massart S., De Meirleir K. (2013). High-throughput 16S rRNA gene sequencing reveals alterations of intestinal microbiota in myalgic encephalomyelitis/chronic fatigue syndrome patients. Anaerobe.

[125] Naseribafrouei A., Hestad K., Avershina E., Sekelja M., Linløkken A., Wilson R., Rudi K. (2014). Correlation between the human fecal microbiota and depression. Neurogastroenterol. Motil..

[126] Maynard C.L., Elson C.O., Hatton R.D., Weaver C.T. (2015). Reciprocal interactions of the intestinal microbiota and immune system. Nature.

[127] Severance E.G., Gressitt K.L., Stallings C.R., Katsafans E., Schweinfurth L.A., Savage C.L., Adamos M.B., Sweeney K.M., Origoni A.E., Khushalani S. (2016). Candida albicans exposures, sex specificity and cognitive deficits in schizophrenia and bipolar disorder. NPJ Schizophr..

[128] Dickerson F., Severance E., Yolken R. (2017). The microbiome, immunity, and schizophrenia and bipolar disorder. Brain Behav. Immun..

[129] Macedo D., Filho A., Soares de Sousa C.N., Quevedo J., Barichello T., Júnior H., Freitas de Lucena D. (2017). Antidepressants, antimicrobials or both? Gut microbiota dysbiosis in depression and possible implications of the antimicrobial effects of antidepressant drugs for antidepressant effectiveness. J. Affect. Disord..

[130] American Psychiatric Association (2000). Diagnostic and Statistical Manual of Mental Disorders.

[131] Nishino R., Mikami K., Takahashi H., Tomonaga S., Furuse M., Hiramoto T., Aiba Y., Koga Y., Sudo N. (2013). Commensal microbiota modulate murine behaviors in a strictly contamination-free environment confirmed by culture-based methods. Neurogastroenterol. Motil..

[132] Kantak P.A., Bobrow D.N., Nyby J.G. (2014). Obsessive-compulsive-like behaviors in house mice are attenuated by a probiotic (Lactobacillus rhamnosus GG). Behav. Pharmacol..

[133] Savignac H.M., Kiely B., Dinan T.G., Cryan J.F. (2014). Bifidobacteria exert strain-specific effects on stress-related behavior and physiology in BALB/c mice. Neurogastroenterol. Motil..

[134] National Institute for Clinical Excellence (NICE) (2005). Obsessive-Compulsive Disorder (OCD) and Body Dysmorphic Disorder (BDD).

[135] Sanikhani N.S., Modarressi M.H., Jafari P., Vousooghi N., Shafei S., Akbariqomi M., Heidari R., Lavasani P.S., Yazarlou F., Motevaseli E. (2020). The effect of Lactobacillus casei consumption in improvement of obsessive-compulsive disorder: An animal study. Probiotics Antimicrob. Proteins.

[136] Kobliner V., Mumper E., Baker S.M. (2018). Reduction in obsessive compulsive disorder and self-injurious behavior with Saccharomyces boulardii in a child with autism: A case report. Integr. Med. (Encinitas).

[137] Altemus M., Pigott T., Kalogeras K.T., Demitrack M., Dubbert B.M., Murphy D.L., Gold P.W. (1992). Abnormalities in the regulation of vasopressin and corticotropin releasing factor secretion in obsessive-compulsive disorder. Arch. Gen. Psychiatry.

[138] Monteleone P., Catapano F., Tortorella A., Maj M. (1997). Cortisol response to d-fenfluramine in patients with obsessive-compulsive disorder and in healthy subjects: Evidence for a gender-related effect. Neuropsychobiology.

[139] Ullrich M., Weber M., Post A.M., Popp S., Grein J., Zechner M., Guerrero González H., Kreis A., Schmitt A.G., Üçeyler N. (2018). OCD-like behavior is caused by dysfunction of thalamo-amygdala circuits and upregulated TrkB/ERK-MAPK signaling as a result of SPRED2 deficiency. Mol. Psychiatry.

[140] Rees J.C. (2014). Obsessive-compulsive disorder and gut microbiota dysregulation. Med. Hypotheses.

[141] Tannock G.W., Savage D.C. (1974). Influences of dietary and environmental stress on microbial populations in the murine gastrointestinal tract. Infect. Immun..

[142] Andersson H., Tullberg C., Ahrné S., Hamberg K., Lazou Ahrén I., Molin G., Sonesson M., Håkansson Å. (2016). Oral administration of *Lactobacillus plantarum* 299v reduces cortisol levels in luman saliva during examination induced stress: A randomized, double-blind controlled trial. Int. J. Microbiol..

[143] Swedo S.E., Leonard H.L., Garvey M., Mittleman B., Allen A.J., Perlmutter S., Lougee L., Dow S., Zamkoff J., Dubbert B.K. (1998). Pediatric autoimmune neuropsychiatric disorders associated with streptococcal infections: Clinical description of the first 50 cases. Am. J. Psychiatry.

[144] Munoz-Bellido J.L., Munoz-Criado S., Garcìa-Rodrìguez J.A. (2000). Antimicrobial activity of psychotropic drugs: Selective serotonin reuptake inhibitors. Int. J. Antimicrob. Agents.

[145] Marder S.R., Cannon T.D. (2019). Schizophrenia. N. Engl. J. Med..

[146] Uher R., Zwicker A. (2017). Etiology in psychiatry: Embracing the reality of poly-gene-environmental causation of mental illness. World Psychiatry.

[147] Khandaker G.M., Zimbron J., Lewis G., Jones P.B. (2013). Prenatal maternal infection, neurodevelopment and adult schizophrenia: A systematic review of population-based studies. Psychol. Med..

[148] Khandaker G.M., Zammit S., Lewis G., Jones P.B. (2014). A population-based study of atopic disorders and inflammatory markers in childhood before psychotic experience in adolescence. Schizophr. Res..

[149] Lachance L.R., McKenzie K. (2014). Biomarkers of gluten sensitivity in patients with non-affective psychosis: A meta-analysis. Schizophr. Res..

[150] Estes M.L., McAllister A.K. (2017). Maternal immune activation: Implications for neuropsychiatric disorders. Science.

[151] Birnbaum R., Jaffe A.E., Chen Q., Shin J.H., Kleinman J.E., Hyde T.M., Weinberger D.R. (2017). Investigating the neuroimmunogenic architecture of schizophrenia. Mol. Psychiatry.

[152] Goldsmith D.R., Rapaport M.H., Miller B.J. (2016). A meta-analysis of blood cytokine network alterations in psychiatric patients: Comparisons between schizophrenia, bipolar disorder and depression. Mol. Psychiatry.

[153] García-Bueno B., Bioque M., Mac-Dowell K.S., Barcones M.F., Martínez-Cengotitabengoa M., Pina-Camacho L., Rodríguez-Jiménez R., Sáiz P.A., Castro C., Lafuente A. (2014). Pro-/anti-inflammatory dysregulation in patients with first episode of psychosis: Toward an integrative inflammatory hypothesis of schizophrenia. Schizophr. Bull..

[154] García-Bueno B., Bioque M., Mac-Dowell K.S., Santabárbara J., Martínez-Cengotitabengoa M., Moreno C., Sáiz P.A., Berrocoso E., Gassó P., Fe Barcones M. (2015). Pro-/antiinflammatory dysregulation in early psychosis: Results from a 1-year follow-up study. Int. J. Neuropsychopharmacol..

[155] Schizophrenia Working Group of the Psychiatric Genomics Consortium (2014). Biological insights from 108 schizophrenia-associated genetic loci. Nature.

[156] Leboyer M., Oliveira J., Tamouza R., Groc L. (2016). Is it time for immunopsychiatry in psychotic disorders?. Psychopharmacology.

[157] Gumusoglu S.B., Stevens H.E. (2018). Maternal inflammation and neurodevelopmental programming: A review of preclinical outcomes and implications for translational psychiatry. Biol. Psychiatry.

[158] Golofast B., Vales K. (2020). The connection between microbiome and schizophrenia. Neurosci. Biobehav. Rev..

[159] Schwarz E., Maukonen J., Hyytiäinen T., Kieseppä T., Orešič M., Sabunciyan S., Mantere O., Saarela M., Yolken R., Suvisaari J. (2018). Analysis of microbiota in first episode psychosis identifies preliminary associations with symptom severity and treatment response. Schizophr. Res..

[160] Zheng P., Zeng B., Liu M., Chen J., Pan J., Han Y., Liu Y., Cheng K., Zhou C., Wang H. (2019). The gut microbiome from patients with schizophrenia modulates the glutamate-glutamine-GABA cycle and schizophrenia-relevant behaviors in mice. Sci. Adv..

[161] Shen Y., Xu J., Li Z., Huang Y., Yuan Y., Wang J., Zhang M., Hu S., Liang Y. (2018). Analysis of gut microbiota diversity and auxiliary diagnosis as a biomarker in patients with schizophrenia: A cross-sectional study. Schizophr. Res..

[162] Yuan X., Zhang P., Wang Y., Liu Y., Li X., Kumar B.U., Hei G., Lv L., Huang X.F., Fan X. (2018). Changes in metabolism and microbiota after 24-week risperidone treatment in drug naïve, normal weight patients with first episode schizophrenia. Schizophr. Res..

[163] Ma X., Asif H., Dai L., He Y., Zheng W., Wang D., Ren H., Tang J.M., Li C., Jin K. (2020). Alteration of the gut microbiome in first-episode drug-naïve and chronic medicated schizophrenia correlate with regional brain volumes. J. Psych. Res..

[164] Khodaie-Ardakani M.R., Mirshafiee O., Farokhnia M., Tajdini M., Hosseini S.M., Modabbernia A., Rezaei F., Salehi B., Yekehtaz H., Ashrafi M. (2014). Minocycline add-on to risperidone for treatment of negative symptoms in patients with stable schizophrenia: Randomized double-blind placebo-controlled study. Psychiatry Res..

[165] Kao A.C., Spitzer S., Anthony D.C., Lennox B., Burnet P. (2018). Prebiotic attenuation of olanzapine-induced weight gain in rats: Analysis of central and peripheral biomarkers and gut microbiota. Transl. Psychiatry.

[166] Pełka-Wysiecka J., Kaczmarczyk M., Bąba-Kubiś A., Liśkiewicz P., Wroński M., Skonieczna-Żydecka K., Marlicz W., Misiak B., Starzyńska T., Kucharska-Mazur J. (2019). Analysis of gut microbiota and their metabolic potential in patients with schizophrenia treated with olanzapine: Results from a six-week observational prospective cohort study. J. Clin. Med..

[167] Seeman M.V. (2020). The gut microbiome and treatment-resistance in schizophrenia. Psychiatr Q..

[168] Ng Q.X., Soh A., Venkatanarayanan N., Ho C., Lim D.Y., Yeo W.S. (2019). A systematic review of the effect of probiotic supplementation on schizophrenia symptoms. Neuropsychobiology.

[169] Roberfroid M.B. (2005). Introducing inulin-type fructans. Br. J. Nutr..

[170] Guo L., Xiao P., Zhang X.K., Yang Y., Yang M., Wang T., Lu H., Tian H., Wang H., Liu J. (2021). Inulin ameliorates schizophrenia via modulation of the gut microbiota and anti-inflammation in mice. Food Funct..

[171] Li S., Song J., Ke P., Kong L., Lei B., Zhou J., Huang Y., Li H., Li G., Chen J. (2021). The gut microbiome is associated with brain structure and function in schizophrenia. Sci. Rep..

[172] Yuan X., Kang Y., Zhuo C., Huang X.F., Song X. (2019). The gut microbiota promotes the pathogenesis of schizophrenia via multiple pathways. Biochem. Biophys. Res. Commun..

[173] Stilling R.M., Dinan T.G., Cryan J.F. (2014). Microbial genes, brain & behaviour-epigenetic regulation of the gut-brain axis. Genes Brain Behav..

[174] Zhou J., Park C.Y., Theesfeld C.L., Wong A.K., Yuan Y., Scheckel C., Fak J.J., Funk J., Yao K., Tajima Y. (2019). Whole-genome deep learning analysis identifies contribution of noncoding mutations to autism risk. Nat. Genet..

[175] Horvath K., Papadimitriou J.C., Rabsztyn A., Drachenberg C., Tildon J.T. (1999). Gastrointestinal abnormalities in children with autistic disorder. J. Pediatr..

[176] White J.F. (2003). Intestinal pathophysiology in autism. Exp. Biol. Med..

[177] Williams B.L., Hornig M., Buie T., Bauman M.L., Cho Paik M., Wick I., Bennetta A., Jabado O., Hirschberg D.L., Lipkin W.I. (2011). Impaired carbohydrate digestion and transport and mucosal dysbiosis in the intestines of children with autism and gastrointestinal disturbances. PLoS ONE.

[178] Berding K., Donovan S.M. (2016). Microbiome and nutrition in autism spectrum disorder: Current knowledge and research needs. Nutr. Rev..

[179] Srikantha P., Mohajeri M.H. (2019). The possible role of the microbiota-gut-brain-axis in autism spectrum disorder. Int. J. Mol. Sci..

[180] Settanni C.R., Bibbò S., Ianiro G., Rinninella E., Cintoni M., Mele M.C., Cammarota G., Gasbarrini A. (2021). Gastrointestinal involvement of autism spectrum disorder: Focus on gut microbiota. Expert Rev. Gastroenterol. Hepatol..

[181] McElhanon B.O., McCracken C., Karpen S., Sharp W.G. (2014). Gastrointestinal symptoms in autism spectrum disorder: A meta-analysis. Pediatrics.

[182] Adams J.B., Johansen L.J., Powell L.D., Quig D., Rubin R.A. (2011). Gastrointestinal flora and gastrointestinal status in children with autism--comparisons to typical children and correlation with autism severity. BMC Gastroenterol..

[183] Kang D.W., Adams J.B., Gregory A.C., Borody T., Chittick L., Fasano A., Khoruts A., Geis E., Maldonado J., Mcdonough-Means S. (2017). Microbiota transfer therapy alters gut ecosystem and improves gastrointestinal and autism symptoms: An open-label study. Microbiome.

[184] Finegold S.M. (2011). State of the art; microbiology in health and disease. Intestinal bacterial flora in autism. Anaerobe.

[185] Li Q., Han Y., Dy A.B.C., Hagerman R.J. (2017). The gut microbiota and autism spectrum disorders. Front. Cell. Neurosci..

[186] Liu F., Li J., Wu F., Zheng H., Peng Q., Zhou H. (2019). Altered composition and function of intestinal microbiota in autism spectrum disorders: A systematic review. Transl. Psychiatry.

[187] Finegold S.M., Molitoris D., Song Y., Liu C., Vaisanen M.L., Bolte E., McTeague M., Sandler R., Wexler H., Marlowe E.M. (2002). Gastrointestinal microflora studies in late-onset autism. Clin. Infect. Dis..

[188] Song Y., Liu C., Finegold S.M. (2004). Real-time PCR quantitation of clostridia in feces of autistic children. Appl. Environ. Microbiol..

[189] Parracho H.M., Bingham M.O., Gibson G.R., McCartney A.L. (2005). Differences between the gut microflora of children with autistic spectrum disorders and that of healthy children. J. Med. Microbiol..

[190] Finegold S.M., Dowd S.E., Gontcharova V., Liu C., Henley K.E., Wolcott R.D., Young E., Summanen P.H., Granpeesheh D., Dixon D. (2010). Pyrosequencing study of fecal microflora of autistic and control children. Anaerobe.

[191] Wang L., Christophersen C.T., Sorich M.J., Gerber J.P., Angley M.T., Conlon M.A. (2011). Low relative abundances of the mucolytic bacterium Akkermansia muciniphila and Bifidobacterium spp. in feces of children with autism. Appl. Environ. Microbiol..

[192] De Angelis M., Piccolo M., Vannini L., Siragusa S., De Giacomo A., Serrazzanetti D.I., Cristofori F., Guerzoni M.E., Gobbetti M., Francavilla R. (2013). Fecal microbiota and metabolome of children with autism and pervasive developmental disorder not otherwise specified. PLoS ONE.

[193] Kang D.W., Park J.G., Ilhan Z.E., Wallstrom G., Labaer J., Adams J.B., Krajmalnik-Brown R. (2013). Reduced incidence of Prevotella and other fermenters in intestinal microflora of autistic children. PLoS ONE.

[194] Wang L., Christophersen C.T., Sorich M.J., Gerber J.P., Angley M.T., Conlon M.A. (2013). Increased abundance of Sutterella spp. and Ruminococcus torques in feces of children with autism spectrum disorder. Mol. Autism.

[195] Strati F., Cavalieri D., Albanese D., De Felice C., Donati C., Hayek J., Jousson O., Leoncini S., Renzi D., Calabrò A. (2017). New evidences on the altered gut microbiota in autism spectrum disorders. Microbiome.

[196] Gondalia S.V., Palombo E.A., Knowles S.R., Cox S.B., Meyer D., Austin D.W. (2012). Molecular characterisation of gastrointestinal microbiota of children with autism (with and without gastrointestinal dysfunction) and their neurotypical siblings. Autism Res..

[197] Cermak S.A., Curtin C., Bandini L.G. (2010). Food selectivity and sensory sensitivity in children with autism spectrum disorders. J. Am. Diet. Assoc..

[198] Sharp W.G., Postorino V., McCracken C.E., Berry R.C., Criado K.K., Burrell T.L., Scahill L. (2018). Dietary intake, nutrient status, and growth parameters in children with autism spectrum disorder and severe food selectivity: An electronic medical record review. J. Acad. Nutr. Diet..

[199] Yule S., Wanik J., Holm E.M., Bruder M.B., Shanley E., Sherman C.Q., Fitterman M., Lerner J., Marcello M., Parenchuck N. (2021). Nutritional deficiency disease secondary to ARFID symptoms associated with autism and the broad autism phenotype: A qualitative systematic review of case reports and case series. J. Acad. Nutr. Diet..

[200] Mayer E.A., Padua D., Tillisch K. (2014). Altered brain-gut axis in autism: Comorbidity or causative mechanisms?. Bioessays.

[201] Sandler R.H., Finegold S.M., Bolte E.R., Buchanan C.P., Maxwell A.P., Väisänen M.L., Nelson M.N., Wexler H.M. (2000). Short-term benefit from oral vancomycin treatment of regressive-onset autism. J. Child. Neurol..

[202] Cenit M.C., Nuevo I.C., Codoñer-Franch P., Dinan T.G., Sanz Y. (2017). Gut microbiota and attention deficit hyperactivity disorder: New perspectives for a challenging condition. Eur. Child. Adolesc. Psychiatry.

[203] Santonicola A., Gagliardi M., Guarino M., Siniscalchi M., Ciacci C., Iovino P. (2019). Eating disorders and gastrointestinal diseases. Nutrients.

[204] Seitz J., Belheouane M., Schulz N., Dempfle A., Baines J.F., Herpertz-Dahlmann B. (2019). The impact of starvation on the microbiome and gut-brain interaction in anorexia nervosa. Front. Endocrinol.

[205] Seitz J., Trinh S., Herpertz-Dahlmann B. (2019). The microbiome and eating disorders. Psychiatr. Clin. N. Am..

[206] Himmerich H., Treasure J. (2018). Psychopharmacological advances in eating disorders. Expert Rev. Clin. Pharmacol..

