# Correction: Marazziti et al. The Microbiota/Microbiome and the Gut–Brain Axis: How Much Do They Matter in Psychiatry? *Life* 2021, *11*, 760

**DOI:** 10.3390/life12070991

**Published:** 2022-07-04

**Authors:** Donatella Marazziti, Beatrice Buccianelli, Stefania Palermo, Elisabetta Parra, Alessandro Arone, Maria Francesca Beatino, Lucia Massa, Barbara Carpita, Filippo M. Barberi, Federico Mucci, Liliana Dell’Osso

**Affiliations:** 1 Department of Clinical and Experimental Medicine Section of Psychiatry, University of Pisa, 56100 Pisa, Italy; beatriceb.90@live.com (B.B.); stefania.palermo@hotmail.com (S.P.); elisabettaparra@virgilio.it (E.P.); alessandroarone@libero.it (A.A.); mariaf.beatino@gmail.com (M.F.B.); massa.lucia116@gmail.com (L.M.); barbara.carpita1986@gmail.com (B.C.); fil.barberi@gmail.com (F.M.B.); liliana.dellosso@med.unipi.it (L.D.); 2 Unicamillus—Saint Camillus International University of Medical and Health Sciences, 00131 Rome, Italy; 3 Dipartimento di Biochimica e Biologia Molecolare, University of Siena, 53100 Siena, Italy; federico.mucci@med.unipi.it

The authors of this paper [1] have agreed that they would like to add Liliana Dell’Osso as a co-author, as she conceptualized and planned the paper, supervised its implementation, including the literature selection, and revised, critically revised and edited the draft manuscript.

The updated authorship is listed as below:

Donatella Marazziti, Beatrice Buccianelli, Stefania Palermo, Elisabetta Parra, Alessandro Arone, Maria Francesca Beatino, Lucia Massa, Barbara Carpita, Filippo M. Barberi, Federico Mucci and Liliana Dell’Osso. 

The updated author contribution is as follows:

**Author Contributions:** D.M., B.B., S.P., E.P., L.M., L.D. and F.M. planned the review study. D.M., F.M., B.C., F.M.B., A.A., M.F.B. and L.D. revised all the specific literature. D.M., S.P., E.P. and L.M. wrote the first version of the paper, which was subsequently revised and approved by all authors. All authors have read and agreed to the published version of the manuscript.

The authors apologize for any inconvenience caused and state that the scientific conclusions are unaffected. The original publication has also been updated.
